# Supplementary material for: Role and mechanism of NCAPD3 in promoting malignant behaviors in gastric cancer
Source: Front Pharmacol. 2024 Apr 22;15:1341039. doi: 10.3389/fphar.2024.1341039 (PMC11070777; doi:10.3389/fphar.2024.1341039)
Supplement: Supplementary file 11 [file DataSheet2.ZIP › GSEA/Transcipt factor/my_analysis.Gsea.1599461814734/SCGGAAGY_ELK1_02.html]

Details for gene set SCGGAAGY\_ELK1\_02[GSEA]

|  || Dataset | filtered\_dataset.sample\_info.cls#WT\_versus\_NCAPD3\_MUT |
| Phenotype | sample\_info.cls#WT\_versus\_NCAPD3\_MUT |
| Upregulated in class | WT |
| GeneSet | SCGGAAGY\_ELK1\_02 |
| Enrichment Score (ES) | 0.24147958 |
| Normalized Enrichment Score (NES) | 1.789544 |
| Nominal p-value | 0.0 |
| FDR q-value | 0.32363647 |
| FWER p-Value | 0.983 |
Table: GSEA Results Summary

  

Fig 1: Enrichment plot: SCGGAAGY\_ELK1\_02      
 Profile of the Running ES Score & Positions of GeneSet Members on the Rank Ordered List

  

| SYMBOL | TITLE | RANK IN GENE LIST | RANK METRIC SCORE | RUNNING ES | CORE ENRICHMENT || 1 | 2935 | GSPT1 | 5 | 1.156 | 0.0129 | Yes |
| 2 | 7170 | TPM3 | 24 | 1.018 | 0.0137 | Yes |
| 3 | 7571 | ZNF23 | 25 | 1.018 | 0.0285 | Yes |
| 4 | 144233 | BCDIN3D | 43 | 0.942 | 0.0289 | Yes |
| 5 | 27304 | MOCS3 | 76 | 0.844 | 0.0163 | Yes |
| 6 | 157680 | VPS13B | 78 | 0.838 | 0.0277 | Yes |
| 7 | 9489 | PGS1 | 84 | 0.820 | 0.0357 | Yes |
| 8 | 51643 | TMBIM4 | 129 | 0.773 | 0.0127 | Yes |
| 9 | 79109 | MAPKAP1 | 130 | 0.770 | 0.0239 | Yes |
| 10 | 163882 | CNST | 140 | 0.757 | 0.0279 | Yes |
| 11 | 22879 | MON1B | 151 | 0.735 | 0.0307 | Yes |
| 12 | 7733 | ZNF180 | 154 | 0.728 | 0.0398 | Yes |
| 13 | 79789 | CLMN | 160 | 0.717 | 0.0463 | Yes |
| 14 | 11162 | NUDT6 | 162 | 0.716 | 0.0559 | Yes |
| 15 | 23450 | SF3B3 | 178 | 0.697 | 0.0544 | Yes |
| 16 | 51018 | RRP15 | 192 | 0.685 | 0.0542 | Yes |
| 17 | 3308 | HSPA4 | 194 | 0.685 | 0.0634 | Yes |
| 18 | 7024 | TFCP2 | 204 | 0.671 | 0.0661 | Yes |
| 19 | 4836 | NMT1 | 208 | 0.669 | 0.0735 | Yes |
| 20 | 79922 | MRM1 | 209 | 0.669 | 0.0832 | Yes |
| 21 | 55028 | C17orf80 | 222 | 0.659 | 0.0834 | Yes |
| 22 | 126792 | B3GALT6 | 223 | 0.658 | 0.0930 | Yes |
| 23 | 55172 | DNAAF2 | 224 | 0.657 | 0.1025 | Yes |
| 24 | 146050 | ZSCAN29 | 226 | 0.654 | 0.1113 | Yes |
| 25 | 221504 | ZBTB9 | 237 | 0.647 | 0.1129 | Yes |
| 26 | 5701 | PSMC2 | 244 | 0.636 | 0.1175 | Yes |
| 27 | 55745 | AP5M1 | 246 | 0.634 | 0.1259 | Yes |
| 28 | 25973 | PARS2 | 266 | 0.620 | 0.1201 | Yes |
| 29 | 27102 | EIF2AK1 | 277 | 0.609 | 0.1212 | Yes |
| 30 | 55275 | VPS53 | 278 | 0.609 | 0.1300 | Yes |
| 31 | 80124 | VCPIP1 | 282 | 0.607 | 0.1365 | Yes |
| 32 | 25972 | UNC50 | 297 | 0.593 | 0.1342 | Yes |
| 33 | 538 | ATP7A | 305 | 0.588 | 0.1373 | Yes |
| 34 | 129831 | RBM45 | 310 | 0.587 | 0.1427 | Yes |
| 35 | 84946 | LTV1 | 319 | 0.583 | 0.1450 | Yes |
| 36 | 6715 | SRD5A1 | 325 | 0.578 | 0.1495 | Yes |
| 37 | 55697 | VAC14 | 329 | 0.576 | 0.1555 | Yes |
| 38 | 23660 | ZKSCAN5 | 345 | 0.570 | 0.1521 | Yes |
| 39 | 79184 | BRCC3 | 348 | 0.569 | 0.1588 | Yes |
| 40 | 51535 | PPHLN1 | 361 | 0.560 | 0.1576 | Yes |
| 41 | 10818 | FRS2 | 363 | 0.558 | 0.1650 | Yes |
| 42 | 5718 | PSMD12 | 372 | 0.552 | 0.1667 | Yes |
| 43 | 5930 | RBBP6 | 390 | 0.543 | 0.1614 | Yes |
| 44 | 9815 | GIT2 | 394 | 0.540 | 0.1669 | Yes |
| 45 | 23315 | SLC9A8 | 398 | 0.537 | 0.1724 | Yes |
| 46 | 5814 | PURB | 399 | 0.536 | 0.1802 | Yes |
| 47 | 60526 | LDAH | 403 | 0.535 | 0.1856 | Yes |
| 48 | 147007 | TMEM199 | 410 | 0.533 | 0.1887 | Yes |
| 49 | 25917 | THUMPD3 | 412 | 0.530 | 0.1956 | Yes |
| 50 | 221786 | FAM200A | 417 | 0.525 | 0.2001 | Yes |
| 51 | 4247 | MGAT2 | 432 | 0.513 | 0.1967 | Yes |
| 52 | 741 | ZNHIT2 | 453 | 0.503 | 0.1884 | Yes |
| 53 | 27097 | TAF5L | 461 | 0.498 | 0.1902 | Yes |
| 54 | 25880 | TMEM186 | 462 | 0.498 | 0.1974 | Yes |
| 55 | 10291 | SF3A1 | 466 | 0.493 | 0.2023 | Yes |
| 56 | 79960 | JADE1 | 468 | 0.493 | 0.2086 | Yes |
| 57 | 5429 | POLH | 471 | 0.490 | 0.2142 | Yes |
| 58 | 7109 | TRAPPC10 | 475 | 0.488 | 0.2190 | Yes |
| 59 | 10969 | EBNA1BP2 | 483 | 0.485 | 0.2206 | Yes |
| 60 | 84261 | FBXW9 | 491 | 0.477 | 0.2221 | Yes |
| 61 | 29101 | SSU72 | 496 | 0.475 | 0.2258 | Yes |
| 62 | 1479 | CSTF3 | 500 | 0.474 | 0.2304 | Yes |
| 63 | 6742 | SSBP1 | 501 | 0.472 | 0.2373 | Yes |
| 64 | 3184 | HNRNPD | 508 | 0.470 | 0.2394 | Yes |
| 65 | 10478 | SLC25A17 | 525 | 0.460 | 0.2336 | Yes |
| 66 | 2188 | FANCF | 545 | 0.449 | 0.2254 | Yes |
| 67 | 7334 | UBE2N | 546 | 0.449 | 0.2319 | Yes |
| 68 | 27229 | TUBGCP4 | 556 | 0.442 | 0.2313 | Yes |
| 69 | 84335 | AKT1S1 | 572 | 0.437 | 0.2260 | Yes |
| 70 | 26057 | ANKRD17 | 589 | 0.431 | 0.2198 | Yes |
| 71 | 79027 | ZNF655 | 594 | 0.427 | 0.2229 | Yes |
| 72 | 5567 | PRKACB | 595 | 0.426 | 0.2291 | Yes |
| 73 | 6208 | RPS14 | 604 | 0.421 | 0.2290 | Yes |
| 74 | 7290 | HIRA | 612 | 0.417 | 0.2296 | Yes |
| 75 | 10445 | MCRS1 | 617 | 0.414 | 0.2325 | Yes |
| 76 | 54663 | WDR74 | 628 | 0.408 | 0.2306 | Yes |
| 77 | 10274 | STAG1 | 633 | 0.406 | 0.2334 | Yes |
| 78 | 6197 | RPS6KA3 | 643 | 0.403 | 0.2322 | Yes |
| 79 | 6905 | TBCE | 652 | 0.399 | 0.2318 | Yes |
| 80 | 79269 | DCAF10 | 668 | 0.394 | 0.2259 | Yes |
| 81 | 23212 | RRS1 | 681 | 0.387 | 0.2221 | Yes |
| 82 | 4507 | MTAP | 690 | 0.384 | 0.2215 | Yes |
| 83 | 5190 | PEX6 | 694 | 0.381 | 0.2247 | Yes |
| 84 | 10452 | TOMM40 | 696 | 0.380 | 0.2294 | Yes |
| 85 | 84975 | MFSD5 | 702 | 0.376 | 0.2310 | Yes |
| 86 | 9391 | CIAO1 | 704 | 0.374 | 0.2356 | Yes |
| 87 | 178 | AGL | 711 | 0.368 | 0.2363 | Yes |
| 88 | 6651 | SON | 716 | 0.365 | 0.2385 | Yes |
| 89 | 83743 | GRWD1 | 720 | 0.364 | 0.2415 | Yes |
| 90 | 51000 | SLC35B3 | 740 | 0.355 | 0.2319 | No |
| 91 | 10726 | NUDC | 754 | 0.346 | 0.2268 | No |
| 92 | 23294 | ANKS1A | 768 | 0.339 | 0.2216 | No |
| 93 | 84522 | JAGN1 | 798 | 0.324 | 0.2037 | No |
| 94 | 1973 | EIF4A1 | 804 | 0.319 | 0.2045 | No |
| 95 | 3704 | ITPA | 809 | 0.314 | 0.2059 | No |
| 96 | 9343 | EFTUD2 | 818 | 0.305 | 0.2041 | No |
| 97 | 6558 | SLC12A2 | 839 | 0.276 | 0.1926 | No |
| 98 | 1984 | EIF5A | 846 | 0.259 | 0.1916 | No |
| 99 | 6237 | RRAS | 869 | -0.280 | 0.1786 | No |
| 100 | 7453 | WARS | 900 | -0.325 | 0.1600 | No |
| 101 | 51141 | INSIG2 | 903 | -0.328 | 0.1632 | No |
| 102 | 6789 | STK4 | 915 | -0.345 | 0.1596 | No |
| 103 | 23608 | MKRN1 | 927 | -0.352 | 0.1562 | No |
| 104 | 25901 | CCDC28A | 946 | -0.366 | 0.1475 | No |
| 105 | 11007 | CCDC85B | 953 | -0.373 | 0.1482 | No |
| 106 | 55589 | BMP2K | 1017 | -0.417 | 0.1053 | No |
| 107 | 833 | CARS | 1022 | -0.420 | 0.1082 | No |
| 108 | 5305 | PIP4K2A | 1033 | -0.432 | 0.1067 | No |
| 109 | 10616 | RBCK1 | 1042 | -0.438 | 0.1069 | No |
| 110 | 80315 | CPEB4 | 1053 | -0.448 | 0.1056 | No |
| 111 | 8864 | PER2 | 1109 | -0.483 | 0.0698 | No |
| 112 | 54629 | FAM63B | 1110 | -0.483 | 0.0768 | No |
| 113 | 4194 | MDM4 | 1117 | -0.492 | 0.0793 | No |
| 114 | 51105 | PHF20L1 | 1140 | -0.506 | 0.0696 | No |
| 115 | 7518 | XRCC4 | 1175 | -0.540 | 0.0509 | No |
| 116 | 472 | ATM | 1193 | -0.568 | 0.0460 | No |
| 117 | 83548 | COG3 | 1211 | -0.588 | 0.0413 | No |
| 118 | 2005 | ELK4 | 1235 | -0.612 | 0.0323 | No |
| 119 | 5187 | PER1 | 1254 | -0.641 | 0.0276 | No |
| 120 | 83931 | STK40 | 1281 | -0.669 | 0.0171 | No |
| 121 | 26273 | FBXO3 | 1312 | -0.723 | 0.0042 | No |
| 122 | 10611 | PDLIM5 | 1335 | -0.758 | -0.0019 | No |
| 123 | 1649 | DDIT3 | 1341 | -0.779 | 0.0055 | No |
| 124 | 8867 | SYNJ1 | 1387 | -0.979 | -0.0153 | No |
| 125 | 26959 | HBP1 | 1395 | -1.073 | -0.0051 | No |
| 126 | 8548 | BLZF1 | 1398 | -1.103 | 0.0093 | No |
Table: GSEA details [plain text format]

  

Fig 2: SCGGAAGY\_ELK1\_02      
 Blue-Pink O' Gram in the Space of the Analyzed GeneSet

  

Fig 3: SCGGAAGY\_ELK1\_02: Random ES distribution      
 Gene set null distribution of ES for **SCGGAAGY\_ELK1\_02**
